# Association between serum 25-hydroxyvitamin d and myeloperoxidase: A cross-sectional study of a general population in China

**DOI:** 10.3389/fnut.2022.948691

**Published:** 2022-08-02

**Authors:** Junteng Zhou, Ruicen Li, Ting Bao, Wei Jiang, Yan Huang

**Affiliations:** ^1^Health Management Center, West China Hospital, Sichuan University, Chengdu, China; ^2^Laboratory of Cardiovascular Diseases, Regenerative Medicine Research Center, West China Hospital, Sichuan University, Chengdu, China

**Keywords:** myeloperoxidase, association, cross-sectional study, cardiovascular diseases, 25-dihydroxyvitamin D

## Abstract

**Background:**

Several studies have found a strong association between cardiovascular diseases and myeloperoxidase (MPO) as a marker of oxidative stress. Although the anti-inflammatory effects of vitamin D in adults have been validated, evidence about the relationship between MPO and 25(OH)D is lacking. This study aimed to investigate the relationship between MPO and 25(OH)D in the general Chinese population.

**Methods:**

From November 2018 to August 2019, a total of 6414 subjects were enrolled in a tertiary referral hospital in China, which included 3,122 women and 3,292 men. The dependent and independent variables were MPO and 25(OH)D, respectively. The confounders included age, sex, body mass index, waist-hip ratio, smoking status, alcohol drinking status, calcium, and parathyroid hormone concentration.

**Results:**

In the fully adjusted model, we found that MPO decreased by 0.12 (95% CI −0.16, −0.08), ng/mL for each unit (1 nmol/L) increase in 25(OH)D. When 25(OH) D was divided into quartiles, compared with Q1 (< 41.4 nmol/L), the adjusted beta coefficients (β) of MPO in Q2–Q4 were −2.29 (95% CI, −4.31 to −0.27), −4.76 (95% CI, −6.83 to −2.69), and −6.07 (95% CI, −8.23 to −3.92), respectively (*P* for the trend < 0.0001). When 25(OH) D was divided according to clinical severity, compared with the severely deficient (< 30 nmol/L) s≥ 30, < 50 nmol/L) and sufficient groups (≥ 50 nmol/L) were −2.59 (95% CI, −5.87 to 0.69) and −5.87 (95% CI, −9.17 to −2.57), respectively (*P* for the trend < 0.0001).

**Conclusion:**

After adjusting for age, sex, BMI, waist-hip ratio, smoking status, alcohol status, calcium, and PTH, circulating 25(OH)D was negatively associated with MPO.

## Introduction

The mortality rate of cardiovascular disease remains high in the world. In 2020, Cardiovascular Diseases (CVDs) were responsible for approximately 19.1 million deaths (1). In China, CVDs became the leading cause of all-age disability-adjusted life-years in 2017 (2). Given the heavy burden of CVDs, exploring risk factors and understanding the underlying mechanisms involved in CVDs are crucial to their prevention and potential therapeutic targets.

Previous studies have found that inflammation and oxidative stress contribute to major components of cardiovascular risk (3, 4). Myeloperoxidase (MPO), a member of the heme peroxidases superfamily that stored in leukocytes and macrophages, is a 146 kDa glycosylated homodimer protein that consists of two monomers (5). Upon leukocyte activation, the main function of MPO released from the cells is to produce reactive oxidants, such as hypochlorous acid and hypothiocyanous acid, to exert innate immune, and antibacterial effects (5, 6). Although MPO has an important physiological function, its maladjustment involved in oxidative stress and inflammation can cause severe tissue damage in several diseases (7). Several studies have found a strong association between MPO and CVDs; that is, elevated MPO is a biomarker for the occurrence and progression of atherosclerosis, coronary heart disease, hypertension, heart failure, and stroke (8–11). Additionally, given the inspiring results against CVDs through inhibition of MPO in animal models (12), we may anticipate new therapeutic targets for the prevention and treatment of CVDs. Although knockout/knockdown of MPO gene expression and the use of some pharmacological treatment targeting MPO can exert cardiovascular protection *in vitro* and *in vivo*, more strategies for regulating MPO are needed, especially in the general population (12).

Vitamin D is a fat-soluble steroid hormone that can be synthesized by sunlight or supplemented through diet. In clinical practice, 25(OH)D (circulating 25-dihydroxyvitamin D) is commonly used to assess vitamin D status in an individual (13). There is substantial evidence that a low 25(OH)D status significantly increases the risk of cardiovascular disease (14, 15). Moreover, 25(OH)D significantly correlates negatively with some systemic inflammatory parameters (for example, neutrophil-lymphocyte ratio, monocyte-lymphocyte ratio and C-reactive protein) in patients undergoing coronary angiography (16). More importantly, vitamin D acts as an antioxidant against oxidative stress and inflammation (17). Although some studies on the cardiovascular benefits of vitamin D are controversial, evidence suggests that vitamin D supplementation improves left ventricular function and inflammation in patients with heart failure (18, 19).

As far as we know, although a previous study found a link between vitamin D status and MPO in 66 obese children (20), there has been no exploration of the relationship between MPO and 25(OH)D despite vitamin D’s anti-inflammatory benefits in adults. There is a need to further elucidate the relationship between vitamin D status and MPO in the general population. Therefore, we conducted a cross-sectional study in general populations (6,414 subjects) without cardiovascular events in China, assuming a negative relationship between 25(OH)D and MPO levels.

## Materials and methods

### Study design and participants

**FIGURE 1:**
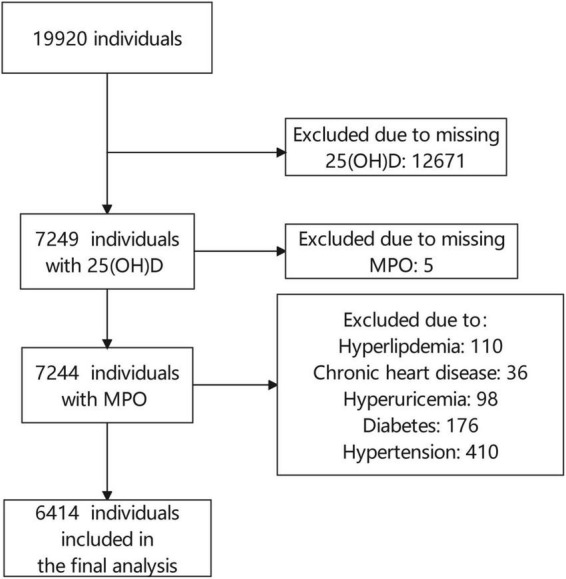
Flow chart of study participants.

This is a population-based cross-sectional study among subjects undergoing routine health examinations at our hospital’s health management center from November 2018 to August 2019. The West China Hospital, a tertiary hospital with three subcenters in Sichuan, provides over 60,000 routine physical examinations annually (21, 22). Participants were enrolled into the study if they fulfilled the following inclusion criteria: voluntarily go to the Health Management Center of West China Hospital of Sichuan University for health examination between November 2018 and August 2019; aged over 18 years; willing to sign an informed consent form. The following criteria demanded their exclusion: (1) incapability to provide informed consent; (2) missing circulating 25(OH)D and MPO measurements; (3) history of hypertension, diabetes, hyperuricemia, chronic heart disease, and hyperlipidemia; and (4) history of use of blood sugar, blood pressure and serum lipid lowering agents. Finally, of the 19,920 consecutive individuals, we excluded those without 25(OH)D and MPO measurements (*n* = 12,676) and those with high-risk factor for cardiovascular diseases (*n* = 830). The study enrolled 6,414 participants in total (Figure 1). The study protocol was approved by the local Ethics Committee of West China Hospital, Sichuan University (No. 2018-303) and informed consent was obtained from all participants. The study was conducted according to the guidelines of the Declaration of Helsinki.

### Demographic data

Demographic and lifestyle information on participants was collected by a trained interviewer through standard procedures as previously reported (21). Specifically, never smoking was defined as self-reported smoking fewer than 100 cigarettes, current smoking was defined as smoking in the past 30 days, and former smoking was defined as not smoking in the past 30 days. Current drinking was defined as one alcohol-unit at least once a week for more than 6 months, former drinking was defined as abstinence from drinking for at least half a year, and never drinking was defined as drinking monthly or less. Self-reported family history of cardiovascular disease was defined as a coronary heart disease, stroke, or peripheral vascular disease in a first-degree relative. Sex, age, smoking, drinking status, self-reported family history of cardiovascular disease, and medical history can be obtained from medical records.

### Anthropometric measurements

Height, weight, waist circumference, and hip circumference were obtained by trained nurses. We measured waist and hip circumference with a flexible and inextensible tape to the nearest 0.1 cm by trained nurses. The waist circumference was measured midway between the anterior superior iliac crest and the 12th costal margin and the hip circumference was measured horizontal around the maximum gluteal circumference in a standing position (23). The body mass index (BMI) was obtained by formula BMI = (weight in kilograms)/(height (in meters)^2^). In accordance with the World Health Organization (WHO), central obesity for the Asian population is defined as a waist-to-hip ratio of more than 0.9 for men and more than 0.8 for women (24).

### Determination of laboratory measurements

After overnight fasting, blood samples were collected into 10 mL EDTA tubes from cubital vein by trained nurses (21). All blood samples were analyzed in strict accordance with standard laboratory test methods in the clinical laboratory of the West China Hospital certified by the China National Accreditation Board.

Serum parathyroid hormone (PTH) concentrations were measured using electrochemiluminescence immunoassays (Cobas^®^8000-e602 modular analyzer, Roche Diagnostics Ltd., Rotkreuz, Switzerland). Serum calcium concentrations were measured on the Cobas 8000-c701 clinical chemistry analyzer. Serum CRP concentrations were measured on a IMMAGE800 analyzer (Beckman Coulter, Inc., United States).

To measure serum 25(OH)D, an enzyme-linked immunosorbent assay (ELISA) was used (Immunodiagnostic Systems, IDS Ltd., London, United Kingdom) as per the manufacturer’s instructions (25). Using a commercial enzyme-linked immunosorbent assay kit (EACHY, Suzhou, China), myeloperoxidase concentrations were determined in plasma samples using standard methods (Supplementary Methods).

### Statistical analysis

Normality of continuous variables was checked by Kolmogorov–Smirnov (KS) test and normal Q-Q plots. For normally distributed continuous variables, the mean ± *SD* is shown; the median and interquartile range (IQR) for non-normally distributed continuous variables are shown. When analyzing normally distributed continuous variables, one-way analysis of variance (ANOVA) with appropriate parametric representation was used. Categorical variables expressed as percentages were compared using the chi-square test. When analyzing non-normally distributed data, Wilcoxon signed ranking was used. As long as variables were categories, McNemar and Yates’s correction tests were performed.

Using an unadjusted and a multivariate-adjusted linear and logistic model, regression coefficient and corresponding 95% confidence intervals (CI) were reported by using unadjusted (crude model), minimally adjusted (adjusted model I), and fully adjusted analysis (adjusted model II) according to STROBE guidelines (26). Specifically, the unadjusted model did not correct for any variables. Model I correct for age (years) and sex. In Model II, age, sex, BMI (kg/m^2^), waist-hip ratio, smoking status, alcohol status, calcium (mmol/L) and PTH (pg/dL) were controlled. To better understand the relationship between MPO and vitamin D, 25(OH)D concentrations were categorized into a categorical variable by quartile and the predefined categories as follows: sufficient (≥ 50 nmol/L), insufficient (30–50 nmol/L), and severely deficient (<30 nmol/L) (27). Furthermore, the non-linear association between 25(OH)D and MPO was explored using a generalized additive model (GAM) model and smooth curve fitting. A sensitivity analysis was conducted by subgroup and interaction analysis to explore the effects of possible modifiers on the 25(OH)D-MPO relationship. When exploring elevated MPO and vitamin D deficiency and insufficiency, we assessed unmeasured confounding by calculating E value (28). The E-value quantifies the required magnitude of an unmeasured confounder that could negate the observed association between MPO and vitamin D deficiency and insufficiency.

Multiple imputation was implemented by chained equations (MICE) to generate five datasets with complete data for missing covariates. Using the standard multiple imputation Rubin’s rules, multivariable and GAM analyses were performed on the combined imputed datasets.

Two-tailed *P*-value < 0.05 was considered statistically significant unless otherwise stated. Statistical analysis was performed using R version 4.0.^1^

## Results

A total of 6,414 subjects were included in the cross-sectional study, which included 3,122 women and 3,292 men. The characteristics of the study participants were grouped into four quantiles, Q1–Q4, depending on the levels of 25(OH)D, as described in Table 1. Between all quintiles of 25(OH)D groups, significant differences were observed in age, sex, BMI, waist-hip ratio, smoking status, alcohol status, calcium, PTH, and MPO. Higher serum 25(OH) D levels were more common in subjects who were older, male, current smokers and drinkers, and had higher serum calcium, lower PTH, and MPO levels. The characteristics of those individuals excluded due to exclusion criteria in the final analysis did not differ substantially from those included (Supplementary Table 1).

**TABLE 1:** Characteristics of the study participants according to serum 25(OH)D concentrations.

		25(OH)D, nmol/L				
		Q1	Q2	Q3	Q4	
	Total	(< 41.4)	(41.41 < 52.0)	(52.0 < 64.6)	(≥ 64.6)	*P*-value
No. of participants	6,414	1,652	1,610	1,583	1,569	
Age (years)	46.45 ± 10.55	44.12 ± 10.46	45.25 ± 10.18	47.05 ± 10.45	49.54 ± 10.31	<0.001
Sex						<0.001
Women	3,122 (48.67%)	1,081 (65.44%)	816 (50.68%)	675 (42.64%)	550 (35.05%)	
Men	3,292 (51.33%)	571 (34.56%)	794 (49.32%)	908 (57.36%)	1,019 (64.95%)	
BMI, kg/m^2^	23.56 ± 3.23	23.29 ± 3.46	23.70 ± 3.30	23.77 ± 3.11	23.47 ± 2.99	<0.001
Waist-hip ratio	0.85 ± 0.07	0.83 ± 0.08	0.85 ± 0.08	0.86 ± 0.07	0.86 ± 0.07	<0.001
Smoking status, *N* (%)						<0.001
Never	4,613 (71.92%)	1,296 (78.45%)	1,177 (73.11%)	1,096 (69.24%)	1,044 (66.54%)	
Former	279 (4.35%)	40 (2.42%)	48 (2.98%)	76 (4.80%)	115 (7.33%)	
Current	1,522 (23.73%)	316 (19.13%)	385 (23.91%)	411 (25.96%)	410 (26.13%)	
Alcohol status, *N* (%)						<0.001
Never	3,567 (55.61%)	1,092 (66.10%)	908 (56.40%)	803 (50.73%)	764 (48.69%)	
Former	52 (0.81%)	7 (0.42%)	14 (0.87%)	12 (0.76%)	19 (1.21%)	
Current	2,795 (43.58%)	553 (33.47%)	688 (42.73%)	768 (48.52%)	786 (50.10%)	
Family history of cardiovascular disease, *N* (%)	285 (4.44%)	77 (4.66%)	63 (3.91%)	71 (4.49%)	74 (4.72%)	0.675
Calcium (mmol/L)	2.33 ± 0.09	2.31 ± 0.09	2.32 ± 0.08	2.33 ± 0.09	2.34 ± 0.08	<0.001
Parathyroid hormone (pg/dL)	6.28 ± 2.07	6.88 ± 2.41	6.40 ± 2.01	6.05 ± 1.84	5.75 ± 1.77	<0.001
CRP (mg/L)	1.91 (1.36–2.89)	1.86 (1.29–2.95)	1.94 (1.38–2.93)	1.91 (1.40–2.82)	1.92 (1.38–2.85)	0.197
25(OH)D, nmol/L	54.42 ± 18.34	34.16 ± 5.66	47.01 ± 3.06	58.35 ± 3.59	79.38 ± 13.82	<0.001
MPO, ng/ml	25.68 (19.06–35.31)	27.37 (21.44–37.21)	26.35 (19.43–36.78)	24.82 (17.48–34.34)	23.77 (17.30–32.76)	<0.001

**FIGURE 2:**
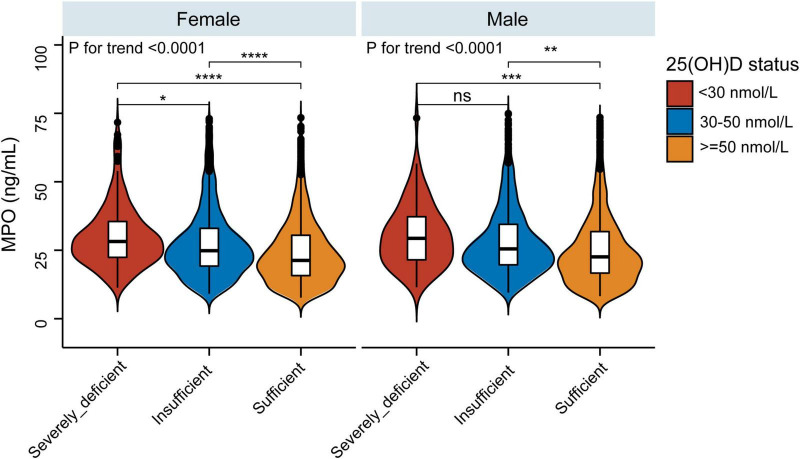
Violin and boxplot representing relative MPO levels between the sufficient, insufficient, and severely deficient 25(OH)D groups. *P < 0.05, **P < 0.01, ***P < 0.001, ****P < 0.0001.

**FIGURE 3:**
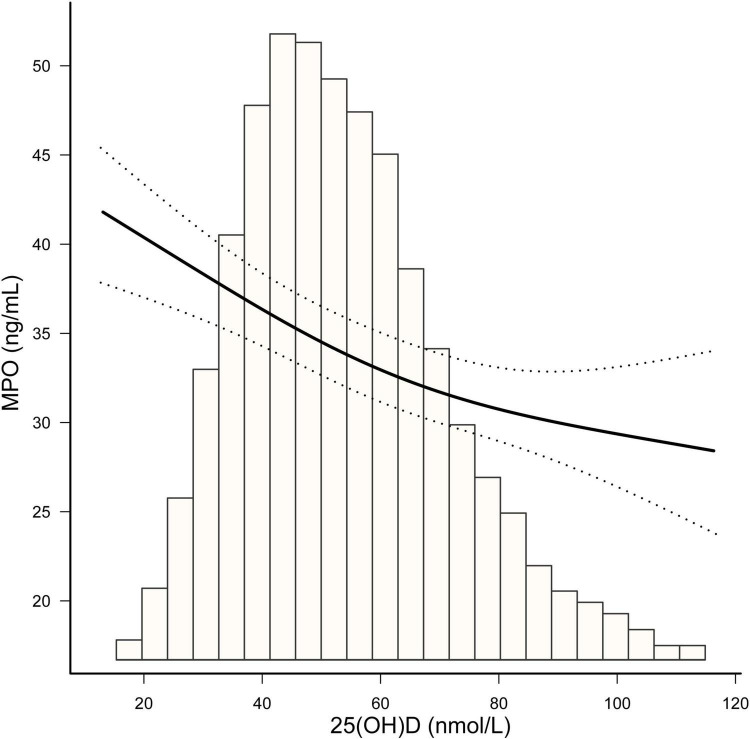
The smooth curve fitting presented linear associations between serum 25(OH)D concentrations and MPO among participants. Adjustment for: age (years), sex, BMI, waist-hip ratio, smoking status, alcohol status, calcium (mmol/L) and parathyroid hormone (pg/dL).

As vitamin D deficiency improved (from severely deficient to insufficient and sufficient 25(OH)D groups), MPO showed a decreasing trend in both men and women (*P* for trend < 0.0001) (Figure 2). The non-linear dose–response curve conducted by GAM demonstrated that the association between 25(OH)D and MPO was linear after adjusting for the confounding variables (Figure 3). Then, the association between 25(OH)D and MPO was observed by univariate and multivariate models, as reported in Table 2. In the crude model, we found that MPO decreased by 0.11 ng/mL for each unit (1 nmol/L) increase in 25(OH)D; the same trend was seen in Model I and Model II after adjusting for other confounding variables. Based on statistical and clinical practice, we then transformed the 25(OH)D level into categorical variables for multivariable analysis as stated in the Methods. There was a strong negative correlation between serum 25(OH) D levels and MPO after controlling for age, sex, BMI, waist-hip ratio, smoking status, alcohol status, calcium and PTH. When 25(OH) D was divided into quartiles, compared with Q1 (< 41.4 nmol/L), the adjusted beta coefficients (β) of MPO in Q2–Q4 were −2.29 (95% CI, −4.31 to −0.27), −4.76 (95% CI, −6.83 to −2.69), and −6.07 (95% CI, −8.23 to −3.92), respectively, with *P* for the trend < 0.0001. When 25(OH) D was divided according to clinical severity, compared with the severely deficient (<30 nmol/L) group, the adjusted beta coefficients (β) of MPO in the insufficient (≥ 30, < 50 nmol/L) and sufficient groups (≥ 50 nmol/L) were −2.59 (95% CI, −5.87 to 0.69) and −5.87 (95% CI, −9.17 to −2.57), respectively, with *P* for the trend < 0.0001.

**TABLE 2:** Effect of 25(OH)D concentrations on MPO.

Variables	*N*	Crude model	Adjusted model I*	Adjusted model II**
		β (95% CI) *P*-value	Adjusted β (95% CI) *P*-value	Adjusted β (95% CI) *P*-value
**25(OH)D, nmol/L**				
**Continuous**	6,414	−0.11 (−0.14, −0.07) < 0.0001	−0.10 (−0.14, −0.06) < 0.0001	−0.12 (−0.16, −0.08) < 0.0001
**Categories**				
< 30	370	Ref	Ref	Ref
≥ 30, < 50	2,533	−1.66 (−4.60, 1.27) 0.2669	−1.63 (−4.57, 1.32) 0.2791	−2.59 (−5.87, 0.69) 0.1215
≥ 50	3,511	−4.80 (−7.68, −1.91) 0.0011	−4.56 (−7.49, −1.62) 0.0023	−5.87 (−9.17, −2.57) 0.0005
P for trend		<0.0001	<0.0001	<0.0001
**Quartiles**				
Q1 (< 41.4)	1,652	Ref	Ref	Ref
Q2 (≥ 41.41, < 52.0)	1,610	−1.67 (−3.51, 0.18) 0.0771	−1.75 (−3.61, 0.10) 0.0641	−2.29 (−4.31, −0.27) 0.0262
Q3 (≥ 52.0, < 64.6)	1,583	−4.01 (−5.86, −2.15) < 0.0001	−4.01 (−5.90, −2.13) < 0.0001	−4.76 (−6.83, −2.69) < 0.0001
Q4 (≥ 64.6)	1,569	−5.28 (−7.14, −3.42) < 0.0001	−5.11 (−7.04, −3.17) < 0.0001	−6.07 (−8.23, −3.92) < 0.0001
P for trend		<0.0001	<0.0001	<0.0001

Adjust I model adjust for: Age (years), sex.

Adjust II model adjust for: Age (years), sex, BMI, Waist-hip ratio, Smoking status, Alcohol status, Calcium (mmol/L) and Parathyroid hormone (pg/dL). The β-values indicate unstandardized regression coefficients. 95% CI indicates 95% confidence interval.

**FIGURE 4:**
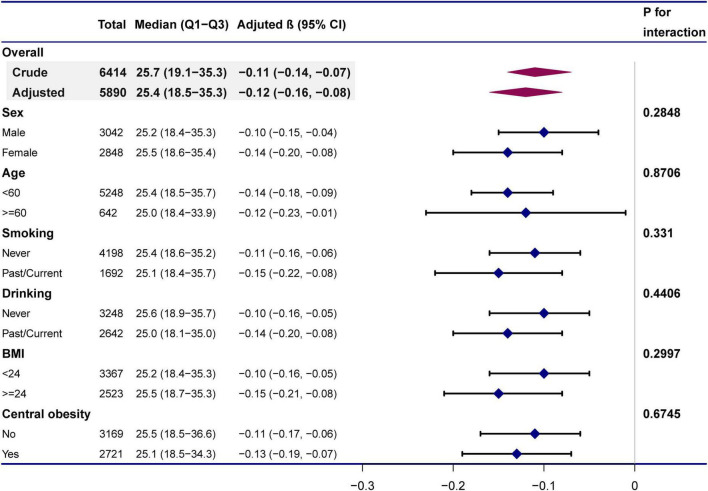
Subgroup analyses of the effect of 25(OH)D concentrations on MPO. Adjustment for: age (years), sex, BMI, waist-hip ratio, smoking status, alcohol status, calcium (mmol/L), and parathyroid hormone (pg/dL) except the stratification variable in each case.

Further assessment of possible moderating factors on the association between 25(OH)D and MPO was achieved through subgroup and interaction analyses. None of the variables, including age (< 60 vs. ≥ 60 years; P for interaction = 0.8706), sex (*P* for interaction = 0.2848), smoking status (Past/Current vs. Never; *P* for interaction = 0.331), drinking status (Past/Current vs. Never; *P* for interaction = 0.4406), BMI (< 24 vs. ≥ 24 kg/m^2^; *P* for interaction = 0.2997), or central obesity (yes vs. no; *P* for interaction = 0.6745), significantly modified the 25(OH)D-MPO relationship (Table 3 and Figure 4).

**TABLE 3:** Effect size of 25(OH)D on MPO in prespecified and exploratory subgroups.

	No of participants	Median (Q1–Q3)	Adjusted β (95% CI)	P for interaction
Sex				0.2848
Male	3,042	25.2 (18.4–35.3)	−0.10 (−0.15, −0.04)	
Female	2,848	25.5 (18.6–35.4)	−0.14 (−0.20, −0.08)	
Age				0.8706
< 60	5,248	25.4 (18.5–35.7)	−0.14 (−0.18, −0.09)	
≥ 60	642	25.0 (18.4–33.9)	−0.12 (−0.23, −0.01)	
Smoke				0.331
Never	4,198	25.4 (18.6–35.2)	−0.11 (−0.16, −0.06)	
Past/Current	1,692	25.1 (18.4–35.7)	−0.15 (−0.22, −0.08)	
Alcohol				0.4406
Never	3,248	25.6 (18.9–35.7)	−0.10 (−0.16, −0.05)	
Past/Current	2,642	25.0 (18.1–35.0)	−0.14 (−0.20, −0.08)	
BMI				0.2997
< 24	3,367	25.2 (18.4–35.3)	−0.10 (−0.16, −0.05)	
≥ 24	2,523	25.5 (18.7–35.3)	−0.15 (−0.21, −0.08)	
Central obesity				0.6745
No	3,169	25.5 (18.5–36.6)	−0.11 (−0.17, −0.06)	
Yes	2,721	25.1 (18.5–34.3)	−0.13 (−0.19, −0.07)	

The β-values indicate unstandardized regression coefficients. 95% CI indicates 95% confidence interval.

To exclude the potential biased effect of missing data, we further performed sensitivity analysis using multiple imputation. As shown in Supplementary Table 2, no significant difference was observed between the created complete data and preimputation data. The relationship between MPO and 25(OH)D was still linear in the pro-imputation data (Supplementary Figure 1). After combining the pro-imputation, we still found a significant negative trend between 25(OH)D and MPO (Supplementary Table 3). After excluding those with thyroid-related diseases (*n* = 31), family history of cardiovascular disease (*n* = 285) and the those with lowest 1% or 2.5% of 25(OH)D levels respectively, the association between 25(OH)D and MPO did not change (Supplementary Tables 4, 5, 7). When MPO was divided into quartiles, Supplementary Table 6 showed that compared with Q1 (<18.35 ng/ml), the adjusted odds ratio (OR) for vitamin D deficiency and insufficiency (25(OH)D < 50 nmol/L) in Q2-Q4 were 1.48 (95% CI, 1.26–1.73), 1.78 (95% CI, 1.52–2.09), and 1.77 (95% CI, 1.51–2.08), respectively, with *P* for the trend < 0.0001. To assess unmeasured confounding by calculating E value, we found that confounders having a relative risk association = 2.94 with both elevated MPO and vitamin D deficiency and insufficiency to deviate our conclusions (Supplementary Figure 2).

## Discussion

Our aim was to investigate whether circulating 25(OH)D was independently associated with MPO. Since the increase in MPO can promote the occurrence and development of cardiovascular diseases and vitamin D is a well-documented protective factor of cardiovascular risk, it is very important to explore the relationship between the two indicators. To our knowledge, the present study is the first population-based report of an independent relationship between 25(OH)D and MPO after adjustment for confounders in Chinese adults.

Numerous studies have linked vitamin D deficiency to metabolic disorders, such as increased levels of inflammation, oxidative stress, reactive oxygen species (ROS) production, insulin resistance, endothelial dysfunction, and disruption of blood sugar and lipids, which contribute to an increased risk of cardiovascular disease (29–31). Recently, Cãtoi et al. (32) conducted a cross-sectional study to investigate the association between 25(OH)D and markers of oxidative stress in 47 patients with type 2 diabetes. They found that compared to those with serum 25(OH)D greater than 20 ng/mL, interleukin 6, total oxidant status and oxidative stress index were significantly higher in the 25(OH)D less than 10 ng/mL and 25(OH)D between 10 and 20 ng/ml group. Codoñer-Franch et al. (20) designed a pioneering observational study to explore the relationship between vitamin D status and MPO in 66 obese Caucasian children from 7 to 14 years old. Consistent with our results, they also found that the MPO in the 25(OH)D insufficient group (<20 ng/m) was higher than that in the 25(OH)D (≥ 20 ng/mL) sufficient group among children. However, the above studies also discussed the relationship between 25(OH)D and other oxidative stress and inflammation indexes. In addition, the small sample size prevented them from formulating a confound-correcting model to satisfy these oxidative stress indicators and 25(OH)D. Specifically, they only adjusted for age, sex and sexual maturity status, but other factors that affect vitamin D metabolism, such as BMI, PTH, lipid levels and calcium levels, should also be considered. However, due to different research focuses, Codoñer-Franch and associates did not discuss the above issues in depth.

Several factors influence the relationship between oxidative stress and vitamin D. According to STROBE guidelines, subgroup and interaction analyses are helpful to reveal the underlying truths (26). Smoking and alcohol consumption have previously been reported to cause oxidative stress through the production of ROS and reactive nitrogen species (RNS) (33, 34). In addition, obesity is associated not only with systemic inflammation and oxidative stress but also with vitamin D deficiency (35–37). A previous study reported significant interactions between acute symptoms and oxidative stress status in patients undergoing coronary angiography (16). In our study, however, we did not find that smoking, alcohol consumption, or obesity affected the MPO-25(OH)D correlation. The reason may be that the population we included was the general population without hypertension, diabetes and hyperlipidemia, in which the risk factors for cardiovascular disease could not be synergistic with oxidative stress.

MPO, a pro-oxidant enzyme, may be a promising target for cardiovascular diseases. Previous studies have shown that MPO-catalyzed nitrification and chlorination can target apolipoprotein A-I (APOA-I), a major component of high-density lipoprotein (HDL) (38). Oxidation of HDL and APOA-I inhibits cholesterol efflux in macrophages, as well as proliferation and migration of vascular smooth muscle cells, resulting in atherosclerotic plaque instability (39, 40). In addition, the activation of MPO can lead to the production of metalloproteinases and the transformation of fibroblasts into myofibroblasts, ultimately contributing to the synthesis, and degradation of collagen (41). It is well established that vitamin D acts as an antioxidant by removing excess ROS. In our study, we found significant inverse correlation between 25(OH)D and MPO, which partly reflects the importance of improvement for vitamin D status. The possible mechanism is that 1,25-dihydroxy vitamin D3 binds to vitamin D receptors to form nuclear receptor-ligand complexes, functioning as transcription factors to regulate the expression of more than 200 genes, including many oxidative stress-related enzymes (42, 43). Consistently, previous studies have also found that vitamin D supplementation significantly increases catalase activity, a hemoprotein evaluation of antioxidant status (44).

Continuing controversy surrounds the range and recommendations for blood concentrations of 25(OH)D. The guidelines from The Institute of Medicine of The National Academies recommend that serum 25(OH)D concentrations greater than 50 nmol/L meet the needs of most people; when the concentration exceeds 125 nmol/L, attention should be given (45). In contrast, according to the Endocrine Society Practice Guidelines, the adequate reference range of sufficient 25(OH)D is 50–250 nmol/L (46). Indeed, in most studies, the upper limit of the safe range for vitamin D is usually defined as 250 nmol/L (47). In our study, 25(OH)D above 125 nmol/L was detected in only 20 individuals. However, 45.2% of the included individuals had 25(OH)D below 50 nmol/L, which shows the importance of vitamin D supplementation in the general population.

Previous meta-analyses have demonstrated that long-term vitamin D supplementation can reduce levels of inflammation and oxidative stress, thereby contributing to cardiovascular protection (48, 49). In a mouse model of periprosthetic joint infection, Hegde et al. (50) found that higher MPO was exhibited in mice fed a vitamin D-deficient diet than in those fed an adequate vitamin D diet. Interestingly, when mice fed a vitamin D-deficient diet were given an adequate vitamin D diet again after surgery, their MPO levels recovered to levels comparable to those of mice on a normal vitamin D diet. Another *in vitro* study indicated that adding vitamin D to the human neutrophil culture medium reduced MPO release by 22% (51). Another interesting observation on the efficacy of vitamin D supplementation on MPO in patients with type 2 diabetes was conducted by Cojic et al. (44). They found that compared with metformin group, vitamin D supplementation plus metformin group resulted in a significant decrease in MPO and a significant increase in antioxidative enzyme activity after 6 months. Therefore, there is reason to believe vitamin D supplementation can lower MPO levels and thus play a cardiovascular protective role in general population. Nevertheless, the mechanisms by which vitamin D affects MPO require further research.

To enhance the level of vitamin D in serum, several exogenous supplementation regimens and lifestyles are recommended for general population. First, since over 90% production of the vitamin D derived from sunshine, it requires short, regular exposures to sunlight without sunscreen (52, 53). However, individuals with darker skin, older age and who live in areas with less sun exposure should consider taking exogenous vitamin D supplementation. Due to the limited supply of vitamin D-fortified foods in most parts of the world, cholecalciferol of 1,000–2,000 IU per day or Ergocalciferol of 50,000 IU per month is recommended for general adults (54, 55).

The strengths of our research are mainly in the following aspects. First, large sample sizes and standardized survey and measurement procedures improve the accuracy and validity of the results. Second, since we focused on exploring the relationship between MPO and vitamin D status, we adopted a more rational strategy for dealing with confounding factors. Third, the GAM model was applied to explore the non-linear relationship in our study. Fourth, subgroup and interaction analyses were performed to further analyze potential factors influencing the relationship between MPO and vitamin D status. Finally, we use multiple imputation to address the impact of missing variables on the results. The above mentioned provides a basis for understanding the mechanism by which vitamin D exerts its protection against oxidative stress from another perspective and for the design of future intervention trials to prevent cardiovascular diseases.

However, our study has some limitations. First, a causal relationship between MPO and vitamin D status cannot be established due to the nature of the cross-sectional study; further long-term follow-up and intervention studies will help provide evidence regarding the effect of vitamin D on MPO. Second, although we corrected for some major confounding factors, bias due to unmeasured confounders was not excluded. We used sensitivity analysis excluding those with thyroid-related diseases, family history of cardiovascular disease and the those with lowest 1% or 2.5% of 25(OH)D levels, respectively, and found that the results of sensitivity analysis did not change primary result (Supplementary Tables 4, 5, 7). Besides, in a sensitivity analysis exploring elevated MPO and vitamin D deficiency and insufficiency, we assessed unmeasured confounding by calculating E value. The results showed that it is unlikely that any unmeasured confounders could explain the association between elevated MPO and vitamin D deficiency/insufficiency (Supplementary Table 6 and Supplementary Figure 2). Third, because we did not include individuals with hypertension, diabetes, hyperlipidemia and cardiovascular diseases, our conclusions cannot be extrapolated to the above-mentioned population. Fourth, in addition to MPO, future studies need to investigate the relationship between vitamin D status and other inflammatory and oxidative stress markers in a large sample of the general population to provide a complete and an overall association and correlation picture. Fifth, the information concerning regular administration of vitamin D supplementation by the participants was not obtained. However, a previous cross-sectional epidemiological survey showed low consumption of vitamin D-related foods (only 18.44% of women consumed more than 250 g of milk) and about 5% women reported taking a vitamin supplement in Sichuan, which reflects low intake of vitamin D supplements (55). In addition, if someone took vitamin D supplementation, there would be fewer cases of vitamin D deficiency/insufficiency, making it more difficult to identify the association between increased MPO and vitamin D deficiency/insufficiency, thus biasing the results toward the null. Of note, the potential resulting from vitamin D supplementation would bias toward to the null and thus result in an underestimation of the association between MPO and vitamin D deficiency/insufficiency.

## Conclusion

Our data have demonstrated that after adjusting for age, sex, BMI, waist-hip ratio, smoking status, alcohol status, calcium and PTH, circulating 25(OH)D is negatively associated with MPO. Further prospective studies and clinical trials are needed to confirm the potential causal relationships.

## Data availability statement

The raw data supporting the conclusions of this article will be made available by the authors, without undue reservation.

## Ethics statement

The studies involving human participants were reviewed and approved by the West China Sichuan University Hospital Research Committee (No. 2018-303). The patients/participants provided their written informed consent to participate in this study.

## Author contributions

JZ collected the data and conducted statistical analyses as well as wrote the manuscript. RL, TB, and WJ involved in fruitful discussions. JZ and YH edited the manuscript. YH manages the entire project. A consensus was reached among all authors regarding the final draft of the manuscript.

## Footnotes

1
http://www.r-project.org/
