# Supplementary material for: Association between serum 25-hydroxyvitamin d and myeloperoxidase: A cross-sectional study of a general population in China
Source: Front Nutr. 2022 Aug 2;9:948691. doi: 10.3389/fnut.2022.948691 (PMC9379339; doi:10.3389/fnut.2022.948691)
Supplement: Supplementary file 1 [file Data_Sheet_1.pdf]

Supplemental Materials

Supplemental Methods

Determination of human MPO concentrations in plasma

Human MPO was prepared and assayed following the manufacturer's directions (EACHY, Suzhou, China). The plasma was centrifuged for 15 minutes at 1000 x g within 30 minutes of collection. Subsequently, the MPO was determined according to the following steps.

1. Prepare all reagents and working standards.
2. Add 100μL of Dilution Buffer to Blank wells.
3. Add 100μL of Standard dilutions in reverse order of serial dilution, samples, or positive control per well. Cover with plate sealer. Incubate for 2 hours on microplate shaker at room temperature.
4. Aspirate each well and wash, repeating the process three times for a total of four washes. Wash by filling each well with 1x Wash Buffer (300μL) using a squirt bottle, manifold dispenser, or autowasher. Complete removal of liquid at each step is essential to good performance. After the last wash, remove any remaining Wash Buffer by aspirating or decanting. Invert the plate and blot it against clean paper towels.
5. Add 100μL of Detection Antibody working solution to each well. Cover with plate sealer. Incubate for 90 minutes on microplate shaker at room temperature.
6. Repeat the aspiration/wash as in step 4.
7. Add 100μL of Streptavidin-HRP Conjugate working solution to each well. Incubate for 45 minutes on microplate shaker at room temperature. Protect from light.
8. Repeat the aspiration/wash as in step 4.
9. Add 100μL of Substrate Solution to each well. Incubate for 3- 7 min on a microplate shaker at room temperature. Protect from light.
10. Add 100μL of Stop Solution to each well. The color in the wells should change from blue to yellow. If the color in the wells is green, or if the color change does not appear uniform, gently tap the plate to ensure thorough mixing.
11. Determine the optical density of each well using a microplate reader set to 450 nm

within 3 minutes.

Supplemental Tables:

Supplementary Table 1. Characteristics of the participants excluded from the analyses and those included in the final analyses

	Excluded	Included
No. of participants	13506	6414
Age (years)	45.26 ± 12.97	46.45 ± 10.55
Sex		
Women	6379 (47.23%)	3122 (48.67%)
Men	7127 (52.77%)	3292 (51.33%)
BMI, kg/m^2	23.47 ± 3.42	23.56 ± 3.23
Waist-hip ratio	0.84 ± 0.08	0.85 ± 0.07
Smoking status, N (%)		
Never	9934 (73.55%)	4613 (71.92%)
Former	630 (4.66%)	279 (4.35%)
Current	2942 (21.78%)	1522 (23.73%)
Alcohol status, N (%)		
Never	7698 (57.00%)	3567 (55.61%)
Former	118 (0.87%)	52 (0.81%)
Current	5690 (42.13%)	2795 (43.58%)
Calcium (mmol/L)	2.33 ± 0.09	2.33 ± 0.09
Parathyroid hormone (pg/dL)	6.53 ± 2.78	6.28 ± 2.07
CRP (mg/L)	3.01 ± 4.56	2.80 ± 4.46
25(OH)D, nmol/L	58.05 ± 19.69	54.42 ± 18.34
MPO, ng/ml	31.71 ± 26.96	31.61 ± 26.97

Supplementary Table 2. Distributions of variables with missing data comparing observed complete case data to results from the datasets with imputed variables from multiple imputation

	Number (%) with missing data	Complete case	Multiple Imputation				
			1	2	3	4	5
BMI, kg/m^2	24 (0.37%)	23.6 ± 3.2	23.6 ± 3.2	23.6 ± 3.2	23.6 ± 3.2	23.6 ± 3.2	23.6 ± 3.2
Waist-hip ratio	32 (0.49%)	0.8 ± 0.1	0.8 ± 0.1	0.8 ± 0.1	0.8 ± 0.1	0.8 ± 0.1	0.8 ± 0.1
Calcium (mmol/L)	471 (7.34%)	2.3 ± 0.1	2.3 ± 0.1	2.3 ± 0.1	2.3 ± 0.1	2.3 ± 0.1	2.3 ± 0.1
Parathyroid hormone (pg/dL)	41 (0.64%)	6.3 ± 2.1	6.3 ± 2.1	6.3 ± 2.1	6.3 ± 2.1	6.3 ± 2.1	6.3 ± 2.1

Supplementary Table 3. Effect of 25(OH)D concentrations on MPO from the datasets with imputed variables from multiple imputation

Variables	Crude model<br>β (95% CI) P value	Adjusted model<br>Adjusted β (95% CI) P value
25(OH)D, nmol/L		
Continuous	-0.11 (-0.14~-0.07)	-0.11 (-0.15~-0.07)
Categories		
<30	Ref	Ref
>=30, <50	-1.66 (-4.6~1.27)	-2.66 (-5.94~0.62)
>=50	-4.8 (-7.68~-1.91)	-5.97 (-9.27~-2.67)
P for trend	<0.0001	<0.0001
Quartiles		
Q1(<41.4)	Ref	Ref
Q2 (41.41<52.0)	-1.78 (-3.64~0.08)	-2.11 (-3.99~-0.23)
Q3(52.0<64.6)	-3.27 (-5.14~-1.41)	-3.72 (-5.64~-1.8)
Q4 (>=64.6)	-5.33 (-7.19~-3.47)	-5.73 (-7.73~-3.73)
P for trend	<0.0001	<0.0001

Adjust model adjust for: Age (years), sex, BMI, Waist-hip ratio, Smoking status, Alcohol status, Calcium (mmol/L) and Parathyroid hormone (pg/dL). The β-values indicate unstandardized regression coefficients. 95% CI indicates 95% confidence interval.

Supplementary Table 4. Effect of 25(OH)D concentrations on MPO after excluding subjects with family history of cardiovascular disease.

Variables	N	Crude model<br>β (95% CI) P value	Adjusted model<br>Adjusted β (95% CI) P value
25(OH)D, nmol/L			
Continuous	6129	-0.11 (-0.14, -0.07) <0.0001	-0.12 (-0.17, -0.08) <0.0001
Categories			
<30	357	Ref	Ref
>=30, <50	2418	-1.98 (-5.01, 1.05) 0.2009	-2.95 (-6.34, 0.43) 0.0869
>=50	3354	-5.07 (-8.05, -2.10) 0.0008	-6.27 (-9.67, -2.86) 0.0003
P for trend		<0.0001	<0.0001
Quartiles			
Q1(<41.74)	1575	Ref	Ref
Q2 (41.74<52.43)	1547	-1.94 (-3.86, -0.03) 0.0461	-2.62 (-4.72, -0.53) 0.0139
Q3(52.43<64.9)	1512	-4.19 (-6.11, -2.27) <0.0001	-5.02 (-7.17, -2.88) <0.0001
Q4 (>=64.9)	1495	-5.48 (-7.40, -3.55) <0.0001	-6.44 (-8.67, -4.20) <0.0001
P for trend		<0.0001	<0.0001

Adjust model adjust for: Age (years), sex, BMI, Waist-hip ratio, Smoking status, Alcohol status, Calcium (mmol/L) and Parathyroid hormone (pg/dL). The β-values indicate unstandardized regression coefficients. 95% CI indicates 95% confidence interval.

Supplementary Table 5. Effect of 25(OH)D concentrations on MPO after excluding subjects with thyroid disorders.

Variables	N	Crude model<br>β (95% CI) P value	Adjusted model<br>Adjusted β (95% CI) P value
25(OH)D, nmol/L			
Continuous	6383	-0.11 (-0.14, -0.07) <0.0001	-0.12 (-0.16, -0.08) <0.0001
Categories			
<30	368	Ref	Ref
>=30, <50	2518	-1.67 (-4.62, 1.28) 0.2669	-2.64 (-5.93, 0.65) 0.1160
>=50	3497	-4.80 (-7.70, -1.91) 0.0012	-5.92 (-9.23, -2.60) 0.0005
P for trend		<0.0001	<0.0001
Quartiles			
Q1(<41.73)	1645	Ref	Ref
Q2 (41.41<52.4)	1596	-1.68 (-3.54, 0.17) 0.0753	-2.32 (-4.34, -0.29) 0.0251
Q3(52.4<65)	1579	-4.02 (-5.88, -2.16) <0.0001	-4.77 (-6.84, -2.69) <0.0001
Q4 (>=65)	1563	-5.32 (-7.18, -3.45) <0.0001	-6.12 (-8.28, -3.96) <0.0001
P for trend		<0.0001	<0.0001

Adjust model adjust for: Age (years), sex, BMI, Waist-hip ratio, Smoking status, Alcohol status, Calcium (mmol/L) and Parathyroid hormone (pg/dL). The β-values indicate unstandardized regression coefficients. 95% CI indicates 95% confidence interval.

Supplementary Table 6. Effect of MPO concentrations on vitamin D deficiency and insufficiency (25(OH)D <50 nmol/L)

Variables	N	Crude model<br>OR (95% CI) P value	Adjusted model<br>Adjusted OR (95% CI) P value
MPO, ng/ml			
Q1(<18.35)	1652	Ref	Ref
Q2 (18.35<25.28)	1610	1.53 (1.33, 1.77) <0.0001	1.48 (1.26, 1.73) <0.0001
Q3(25.28<35.82)	1583	1.82 (1.57, 2.09) <0.0001	1.78 (1.52, 2.09) <0.0001
Q4 (>=35.82)	1569	1.87 (1.62, 2.17) <0.0001	1.77 (1.51, 2.08) <0.0001
P for trend		<0.0001	<0.0001

Adjust model adjust for: Age (years), sex, BMI, Waist-hip ratio, Smoking status, Alcohol status, Calcium (mmol/L) and Parathyroid hormone (pg/dL). The OR indicates odds ratio. 95% CI indicates 95% confidence interval.

Supplementary Table 7. Effect of 25(OH)D concentrations on MPO after excluding subjects with the lowest 1% or 2.5% of 25(OH)D levels.

Exposure	N	Crude model<br>β (95% CI) P value	Adjusted model<br>Adjusted β (95% CI) P value
25(OH)D, nmol/L	6346		
Continuous (>99%)		-0.10 (-0.14, -0.07) <0.0001	-0.12 (-0.16, -0.07) <0.0001
Categories (>99%)			
Q1(<42.07)	1632	Ref	Ref
Q2 (41.41<52.65)	1597	-1.43 (-3.28, 0.43) 0.1312	-2.05 (-4.07, -0.02) 0.0476
Q3(52.65<65.13)	1567	-4.01 (-5.88, -2.15) <0.0001	-4.77 (-6.84, -2.69) <0.0001
Q4 (>=65.13)	1550	-5.03 (-6.90, -3.16) <0.0001	-5.83 (-7.98, -3.67) <0.0001
P for trend		<0.0001	<0.0001
25(OH)D, nmol/L			
Continuous (>97.5%)	6242	-0.10 (-0.14, -0.07) <0.0001	-0.12 (-0.16, -0.07) <0.0001
Categories (>97.5%)			
Q1(<42.6)	1604	Ref	Ref
Q2 (41.41<52.95)	1571	-1.30 (-3.18, 0.58) 0.1752	-1.84 (-3.89, 0.20) 0.0777
Q3(52.95<65.38)	1540	-3.99 (-5.88, -2.11) <0.0001	-4.71 (-6.80, -2.62) <0.0001
Q4 (>=65.38)	1527	-4.87 (-6.77, -2.98) <0.0001	-5.61 (-7.78, -3.44) <0.0001
P for trend		<0.0001	<0.0001

Adjust model adjust for: Age (years), sex, BMI, Waist-hip ratio, Smoking status, Alcohol status, Calcium (mmol/L) and Parathyroid hormone (pg/dL). The β-values indicate unstandardized regression coefficients. 95% CI indicates 95% confidence interval.

Supplemental Figures:

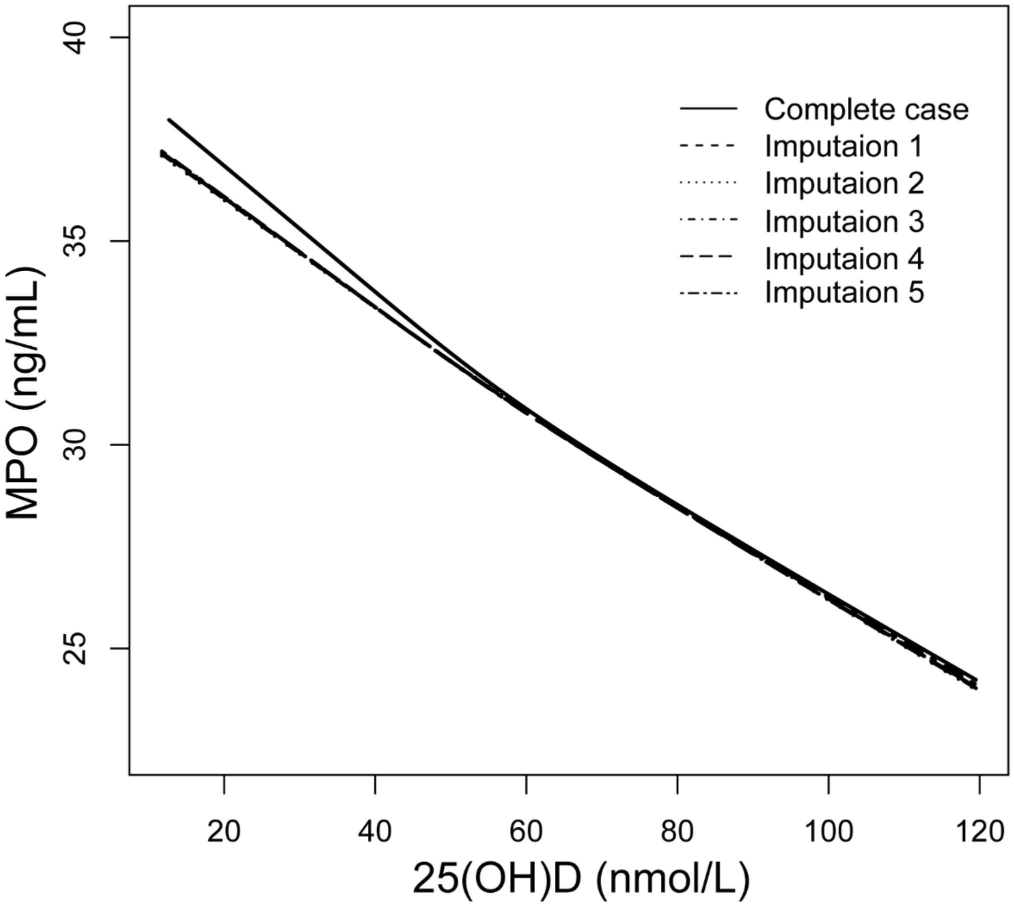

Supplementary Figure 1. The smooth curve fitting presented linear associations between serum 25(OH)D concentrations and MPO from the datasets with imputed variables from multiple imputation. Adjustment for age (years), sex, BMI, waist-hip ratio, smoking status, alcohol status, calcium (mmol/L) and parathyroid hormone (pg/dL).

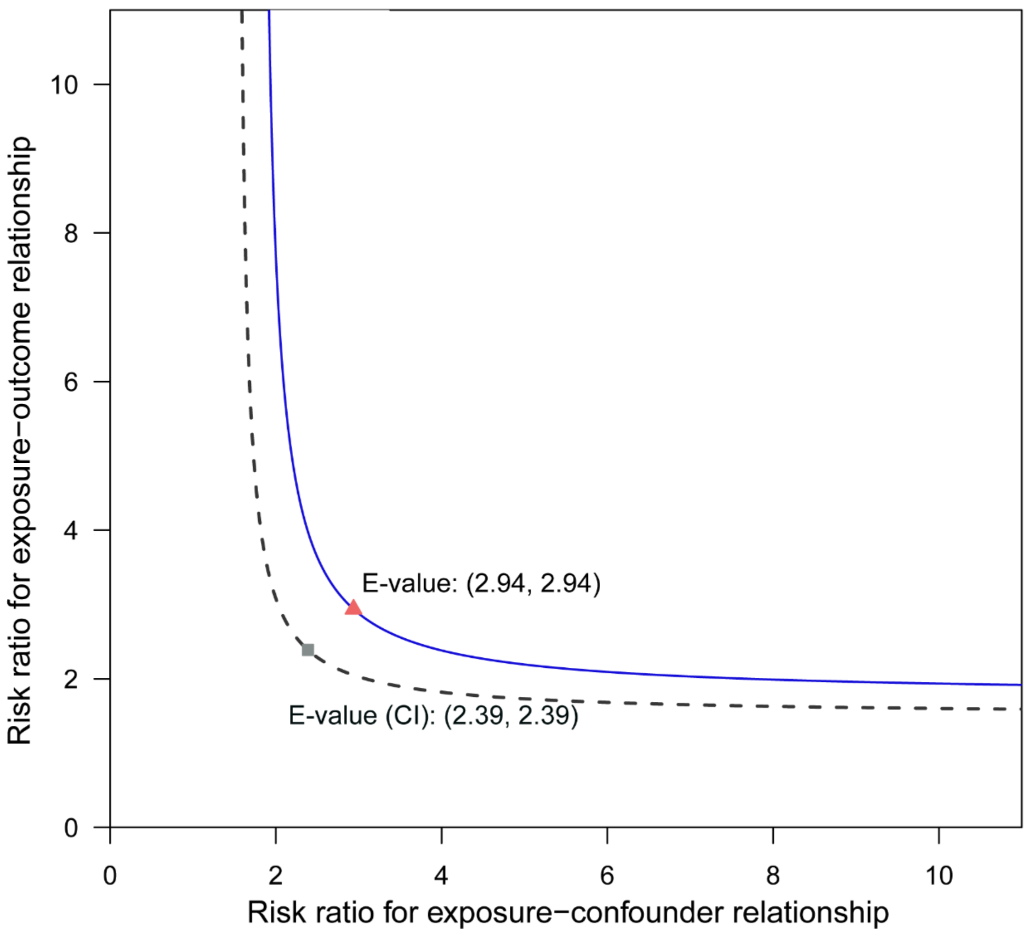

Supplementary Figure 2. Value of the joint minimum strength of association on the risk ratio scale that an unmeasured confounder would be required to have with MPO concentrations (the exposure), and vitamin D deficiency and insufficiency (the outcome).
